# Water, Sanitation, and Public Health

**DOI:** 10.1155/2013/641749

**Published:** 2013-09-24

**Authors:** Niyi Awofeso, Boo Kwa, Stephen Peckham

**Affiliations:** ^1^School of Health and Environmental Studies, Hamdan Bin Mohammed e-University, Dubai, UAE; ^2^Department of Global Health, University of South Florida, MDC 56, Tampa, FL 33612-3805, USA; ^3^Centre for Health Services Studies, University of Kent, George Allen Wing Canterbury, Kent CT2 7NF, UK

*“…We must also consider the qualities of the waters, for as they differ from one another in taste and weight, so also do they differ much in their qualities…”—Hippocrates, On Airs, Waters and Places.*

The organised quest for providing humans with potable water and high quality sanitation has its roots in Hippocratic treatises and heralded the Miasma and Contagion eras of modern Public Health post-1848 British Public Health Act. The 2000 Millennium Development Goal Target 7c seeks to reduce by half (from 24% to 12%), between 1990 and 2015, “the proportion of the population without sustainable access to safe drinking water and basic sanitation.” While current projections indicate that about 9% of the world's population (670 million people) will lack access to potable water by 2015, the achievement of an aspect of this MDG target does not adequately rectify several important problems related to public health aspects of water quality. Similarly, even if the MDG sanitation goal is met by 2015, at least 1.3 billion of currently estimated 2.5 billion people will still be practicing open defecation and making themselves and their neighbours vulnerable to faecal-oral transmission of microbes and unaesthetic environments. Poor sanitation contributes to 1.5 million child deaths every year. This special issue explores many important dimensions of the nexus between “old” public health priorities of water and sanitation from a contemporary public health perspective.

D. Ferguson et al. compared the accuracy of the United States' EPA Method 1600 and Enterolert in measuring faecal contamination of public beaches. They found Enterolert to be superior in detecting nonenterococcus organisms as well as for specific detection of *E. faecalis* in waste water specimens. However, in species identification using culture techniques, EPA Method 1600 was found to be superior—“Horses for Courses.”

Abdelzaher et al. offer policy options for US Environmental Protection Agency's efforts to develop new recreational beaches' monitoring guideline. They proposed a “Comprehensive Toolbox within an Approved Process” (CTBAP) approach, which combines flexibility and consistency and uses mainly human health endpoints of skin disorders and gastrointestinal illness to assure minimum standards of beach water profiles, protect the health of beach swimmers, and limit “overconservatism,” which frequently results in unnecessary beach closures and economic losses.

R. Sauerheber explores the physiologic conditions (such as calcium and pH levels) and systemic effects that of ingested fluoride as well as the efficacy of ingested artificially fluoridated water on dental caries prevention. His paper highlights the important distinction that should be made between naturally occurring fluoride (calcium fluoride CaF_2_) found in water supplies and added fluoride compounds (sodium fluoride NaF and fluorosilicic acid H_2_SiF_6_). Artificial water fluoridation is undertaken in a limited number of countries (mainly the USA, Canada, Ireland, Australia, and New Zealand) as a dental public health intervention aimed at reducing tooth decay. It is a controversial public health measure, and concerns about the effectiveness and safety of fluoridation have been raised for many years [1–3]. This paper specifically examines the physiological effects of artificial fluoridation and why concerns about harm are justified. The analysis is based on a detailed review of the effect of fluorides on physiological functions. The paper sets this discussion within the context of US regulations on the quality of water and responsibilities of regulatory authorities. Sauerheber concludes that there are harmful effects from adding artificial fluoride compounds to water supplies, and in the light of the lack of evidence on effectiveness for reducing dental decay, there are no grounds for continuing policies that force people to ingest fluoride through the consumption of drinking water. Most analyses of fluoridation rarely focus on detailed physiological analysis but rely on observational epidemiological data to demonstrate effectiveness which are rarely sensitive enough or examine potential issues of harm. What is clear is that there are important and urgent questions that public health authorities must address.

Berisha and Gossler conducted a large survey drinking water quality in Kosovo for level of trace elements. The authors highlighted the potential and ongoing adverse consequences of poor monitoring of drinking water, such as persistently raised levels of Arsenic, particularly from private wells.

G. Carrosco-Turiges et al. examined the impact of boiling and filtration modalities on trihalomethane disinfection by-products, some of which are carcinogenic. They found that Bromine-containing trihalomethanes were mostly eliminated when filtering, while chloroform- containing trialomethanes were mostly eliminated by boiling.

K. Al-Bayatti et al. conducted that physicochemical and bacteriological studies on water flowing into consumers' homes form three major water purification stations within Baghdad. They found inefficient purification systems, and possible contamination in distribution system was evidenced by total viable bacterial counts in water of the stations studied of between 1–64 CFU/100 mL. In line with John Snow's 1849–1853 studies in London's Soho district, the authors found that high coliform counts in drinking water supplies correlated closely with high rates of gastroenteritis among children resident in these areas.

As the annual World Toilet Summit continues to grow in profile and efficacy over the past 13 years in focusing on policy makers' attention to the unfinished business of sanitation, and as the United Nations' International Decade for Action on “Water for Life—2005–2015” comes to an end, we hope that readers and policy makers can learn from the findings presented in this special issue to improve the quality of water and sanitation and advance global public health.

*Niyi Awofeso* *Niyi Awofeso* *Boo Kwa* *Boo Kwa* *Stephen Peckham* *Stephen Peckham*
